# Microbial community-scale metabolic modeling predicts personalized short-chain-fatty-acid production profiles in the human gut

**DOI:** 10.1101/2023.02.28.530516

**Published:** 2023-03-01

**Authors:** Nick Bohmann, Tomasz Wilmanski, Lisa Levy, Johanna W. Lampe, Thomas Gurry, Noa Rappaport, Christian Diener, Sean M. Gibbons

**Affiliations:** 1 Institute for Systems Biology, Seattle, WA 98109, USA; 2 Molecular Engineering Graduate Program, University of Washington, Seattle, WA 98195, USA; 3 Fred Hutchinson Cancer Center, Seattle, WA 98109, USA; 4 Pharmaceutical Biochemistry Group, School of Pharmaceutical Sciences, University of Geneva, Switzerland; 5 Myota GmbH, Berlin, Germany; 6 Departments of Bioengineering and Genome Sciences, University of Washington, Seattle, WA 98195, USA; 7 eScience Institute, University of Washington, Seattle, WA 98195, USA

**Keywords:** gut microbiome, short chain fatty acids, flux balance analysis, metabolic model, precision nutrition

## Abstract

Microbially-derived short-chain fatty acids (SCFAs) in the human gut are tightly coupled to host metabolism, immune regulation, and integrity of the intestinal epithelium. However, the production of SCFAs can vary widely between individuals consuming the same diet, with lower levels often associated with disease. A mechanistic understanding of this heterogeneity is lacking. We present a microbial community-scale metabolic modeling (MCMM) approach to predict individual-specific SCFA production profiles. We assess the quantitative accuracy of our MCMMs using *in vitro*, *ex vivo*, and *in vivo* data. Next, we identify associations between MCMM SCFA predictions and a panel of blood-based clinical chemistries in a large human cohort. Finally, we demonstrate how MCMMs can be leveraged to design personalized dietary, prebiotic, and probiotic interventions that optimize SCFA production in the gut. Our results represent an important advance in engineering gut microbiome functional outputs for precision health and nutrition.

## Introduction

The human gut microbiota maintains intestinal barrier function, regulates peripheral and systemic inflammation, and breaks down indigestible dietary components and host substrates into a wide range of bioactive compounds ^1,2^. One of the primary mechanisms by which the gut microbiota impacts human health is through the production of small molecules that enter the circulation and are absorbed and transformed by host tissues ^3–5^. Approximately half of the metabolites detected in human blood are known to be significantly associated with cross-sectional variation in gut microbiome composition ^6^.

Short-chain-fatty-acids (SCFAs) are among the most abundant metabolic byproducts produced by the gut microbiota, largely through the fermentation of indigestible dietary fibers and resistant starches, with acetate, propionate and butyrate being the most abundant SCFAs ^7–9^. Deficits in SCFA production have been repeatedly associated with disease^10,11^. Therefore, SCFA production is a crucial ecosystem service that the gut microbiota provides to its host, with far-reaching impacts on health ^1,11–13^. However, different human gut microbiota provided with the same exact dietary substrate can show variable SCFA production profiles ^14,15^, and predicting this heterogeneity remains a fundamental challenge to the microbiome field. Measuring SCFA abundances in blood or feces is rarely informative of *in situ* production rates, due to the volatility of SCFAs, cross-feeding among microbes, and the rapid consumption and transformation of these metabolites by the colonic epithelium ^10,16,17^. Furthermore, SCFA production fluxes (i.e., the amount of a metabolite produced over a given period of time) within an individual can vary longitudinally, depending upon dietary inputs and the availability of host substrates ^18^. In order to account for this inter- and intra-individual heterogeneity, we propose the use of microbial community-scale metabolic models (MCMMs), which mechanistically account for metabolic interactions between gut microbes, host substrates, and dietary inputs, to estimate personalized, context-specific SCFA production profiles.

Statistical modeling and machine-learning approaches for predicting metabolic output from the microbiome have shown promising results in recent years. For example, postprandial blood glucose responses can be predicted by machine-learning algorithms trained on large human cohorts ^19,20^. However, machine-learning methods are limited by the measurements and interventions represented within the training data ^21^. Mechanistic models like MCMMs, on the other hand, do not rely on training data and provide causal insights ^17^. MCMMs are constructed using existing knowledge bases, including curated genome-scale metabolic models (GEMs) of individual taxa ^22^. MCMMs can be limited by the inability to find well-curated GEMs for abundant taxa present in certain samples, and this underrepresentation in GEMs tends to be worse in human populations that are generally underrepresented in microbiome research ^23^. Despite this, MCMMs can be powerful, transparent, knowledge-driven tools for predicting community-specific responses to a wide array of interventions or perturbations*.*

Here, we demonstrate the utility of MCMMs for the prediction of personalized SCFA production profiles in the context of different dietary, prebiotic, and probiotic inputs. We first validate our modeling platform using synthetic *in vitro* gut microbial communities (N=1,387) and *ex vivo* stool incubation assays (N=21). Next, we investigate the relevance of this modeling strategy *in vivo* using data from a 10-week high-fiber dietary intervention cohort (N=18), where individuals showed a variety of immune responses. We assess the clinical significance of these precision SCFA predictions by looking at associations between predicted SCFA production on an average European diet and a panel of blood-based clinical lab tests in a large human cohort (N=2,687). Finally, we demonstrate the power of MCMMs in designing personalized prebiotic, probiotic, and dietary interventions that optimize individual-specific butyrate production rates.

## Results

### MCMMs capture SCFA production rates in vitro

**Figure 1.**
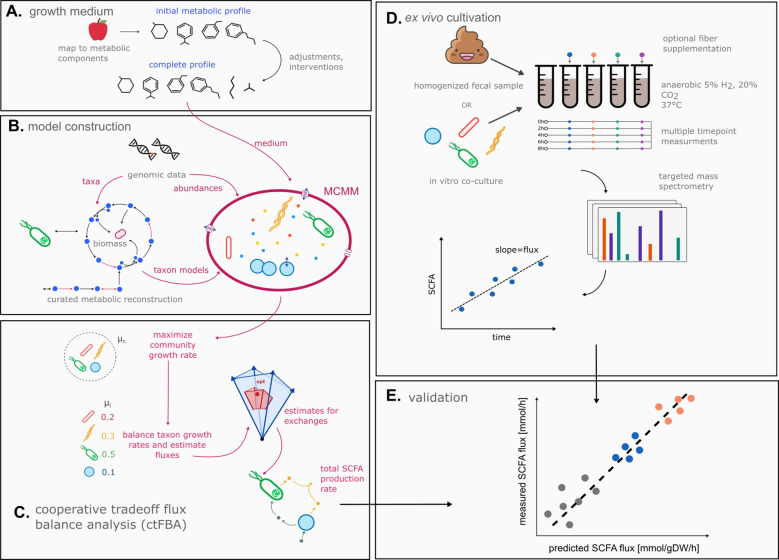
Microbial community-scale metabolic models (MCMMs) predict personalized SCFA production profiles. Schematic of our workflow for validating MCMM-based personalized predictions for SCFA production. **(A)** Prior to modeling, an *in silico* medium is constructed, containing a matched diet mapped to its constituent metabolic components. The medium is depleted in compounds absorbed by the host in the small intestine and augmented with other host-supplied compounds, in addition to adding a minimal set of metabolites required for growth. **(B)** MCMMs are constructed, combining abundance and taxonomic data with pre-curated GEMs into a community model. **(C)** Growth in the MCMM is simulated through cooperative tradeoff flux balance analysis (ctFBA), yielding predicted growth rates and SCFA production fluxes. **(D)** To validate predicted levels of SCFA production fluxes, measured values of production fluxes are collected from fecal samples cultured anaerobically *ex vivo* at 37°C over time. **(E)** Predicted and measured SCFA production fluxes are compared to assess the accuracy of the model.

We sought to investigate whether MCMMs can predict production rates of the major SCFAs (i.e., acetate, propionate, and butyrate) under controlled experimental conditions (Fig. 1). We assembled microbial community models for *in vitro* data sets spanning 4 independent studies with varying levels of complexity. Models were constructed by combining manually-curated GEMs from the AGORA database ^24^, constraining taxon abundances using 16S amplicon sequencing relative abundance estimates, and applying an appropriate growth medium. Sample-specific metabolic models were then solved using cooperative tradeoff flux balance analysis (ctFBA), a previously-reported two-step quadratic optimization strategy that yields empirically-validated estimates of the steady state growth rates and metabolic uptake and secretion fluxes for each taxon in the model ^17^ (see Materials and Methods). Models were summarized at the genus level, which was the finest level of phylogenetic resolution that the 16S data allowed for.

**Figure 2.**
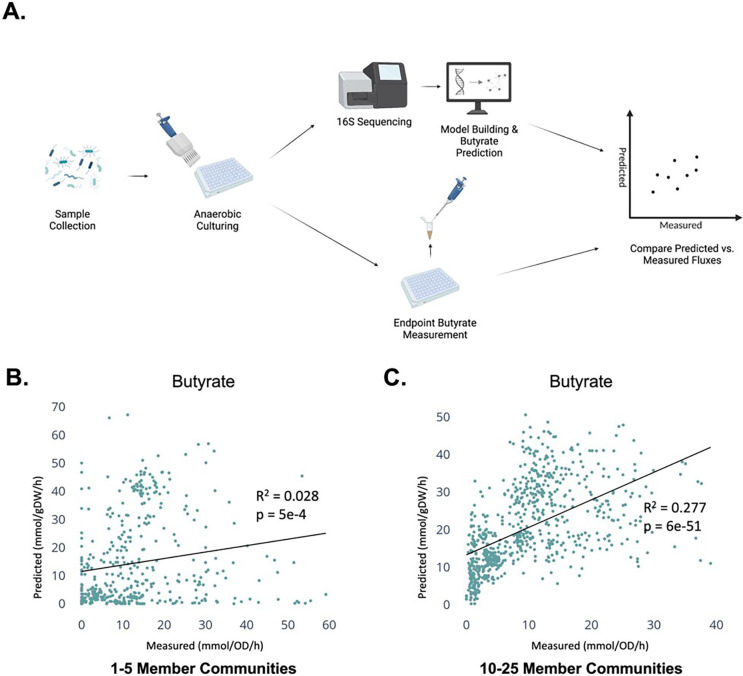
Relationship between predicted and measured butyrate production rates in *in vitro* co-cultures. Each point denotes one of 1,387 anaerobic co-culture assays. Butyrate production flux predictions from MCMMs are shown on the y-axes and measured values are shown on the x-axes, along with R^2^ and p-values from a Pearson’s correlation **(A)** Synthetically constructed communities were cultured anaerobically in a defined medium. Endpoint butyrate concentration was measured and compared with MCMM-predicted flux. **(B)** Predicted and measured butyrate fluxes in models of low richness synthetic communities (1–5 genera per model, N = 882). **(C)** Predicted and measured butyrate fluxes in models of high richness synthetic communities (10–25 genera, N = 697). In (B-C) the dashed line denotes a linear model fit to the data.

First, we looked at data from synthetically constructed *in vitro* cultures of human gut microbial communities obtained from a recent publication ^25^. This data set included measurements of relative microbial abundances, butyrate production levels, and the overall optical density for each of 1,387 independent co-cultures (Fig. 2A). Cultures varied in richness from 1–25 strains. MCMMs were constructed for each co-culture as described above, simulating growth of each of the models using a defined, componentized medium, matching the composition of the medium used in the *in vitro* experiments (see Material and Methods). Model-predicted fluxes of butyrate were compared with measured butyrate production rates normalized to the OD600, stratifying results into low richness (1–5 genera) and high richness (10–25 genera) communities. Model predictions for butyrate production fluxes were significantly associated with measured butyrate production fluxes (Pearson’s correlation; Low Richness: R^2^=0.028, p= 5e-4; High Richness: R^2^=0.277, p= 6e-51), but predictions were more accurate in the higher richness communities (Fig. 2B–C).

**Figure 3.**
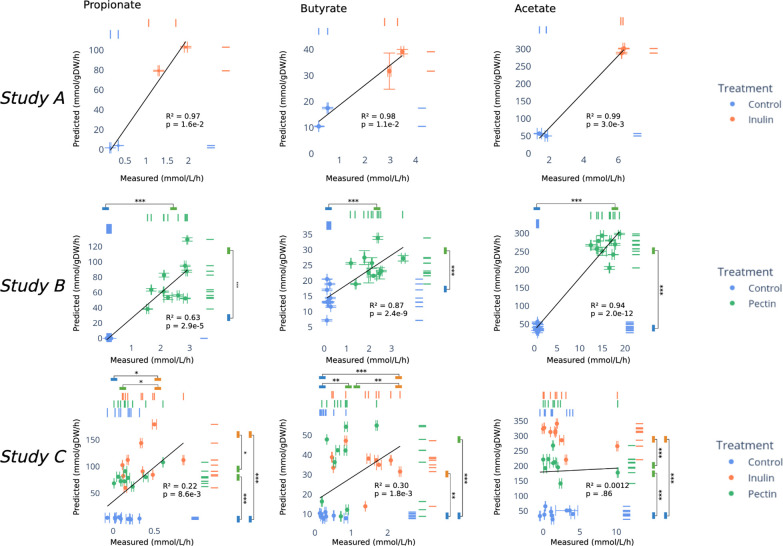
Human stool *ex vivo* assays show quantitative agreement between measured and predicted SCFA production fluxes. SCFA production flux predictions from MCMMs are shown on the y-axes and measured values are shown on the x-axes, along with R^2^ and p-values from a Pearson’s correlation. Marginal rug plots show separation in SCFA production between treatment groups. Error bars show standard error as calculated from measured and predicted values for each sample in triplicate. **(A-C),** Results from a two-donor *ex vivo* study (Study A) showed significant agreement between measured and prediction rates for all three SCFAs following inulin treatment. **(D-F)** Results from Study B^14^, which included pectin treatments to stool homogenates from 10 individuals ^14^. Samples showed significant association between predicted and measured production rates for all three SCFAs. **(G-I)** Results from Study C, which included both pectin and inulin interventions across stool homogenates from 9 individuals. Samples treated with inulin and pectin showed significant associations between predicted and measured fluxes for both propionate and butyrate, but not for acetate.

Next, we compared MCMM predictions to anaerobic *ex vivo* incubations of human stool samples from a small number of individuals (N=21) cultured after supplementation with sterile PBS buffer or with different dietary fibers across three independent studies. Study A contained samples from two donors cultured for 7 hours, Study B ^14^ contained samples from 10 donors cultured for 24 hours, and Study C contained samples from 9 donors cultured for 4 hours. Fecal *ex vivo* assays allow for the direct measurement of bacterial SCFA production fluxes without interference from the host. For all three studies, *ex vivo* incubations were performed by homogenizing fecal material in sterile buffer under anaerobic conditions, adding control or fiber interventions to replicate fecal slurries, and measuring the resulting SCFA production rates *in vitro* at 37°C (see Materials and Methods). Metagenomic or 16S amplicon sequencing data from these *ex vivo* cultures were used to construct MCMMs, using relative abundances obtained from sequencing data as a proxy for relative biomass for each bacterial genus (see Materials and Methods). MCMMs were simulated using a diluted standardized European diet (i.e., to approximate residual dietary substrates still present in the stool slurry), with or without specific fiber amendments, matching the experimental treatments (see Material and Methods). The resulting SCFA flux predictions were then compared to the measured fluxes. We observed an agreement between MCMM-predicted and measured SCFA production fluxes across all three *ex vivo* data sets (Fig. 3). Models predicted significantly higher SCFA production fluxes for fiber-treated samples across all studies (Independent Student’s t-test, p <0.05; Study A was omitted from this analysis due to low sample size, although the separation between controls and fiber-treated samples is visually apparent). The same held true for measured SCFA fluxes, with the exception of acetate in Study C (i.e., the study with the shortest incubation time), where there was not always significant separation in measured SCFA production between control and fiber treatments (Fig. 3G–I). With one exception (Fig. 3J), a significant positive correlation was observed between predicted and measured SCFA fluxes across treatment groups for all three studies (R^2^=0.22–0.99, Pearson test, p<0.05). In summary, we observed agreement between MCMM-predicted and measured *in vitro* SCFA production rates in the presence or absence of fiber supplementation, with better agreement in more diverse communities and over longer experimental incubation times (Fig. 2–3).

### MCMM predictions correspond with variable immunological responses to a 10-week high-fiber dietary intervention

**Figure 4.**
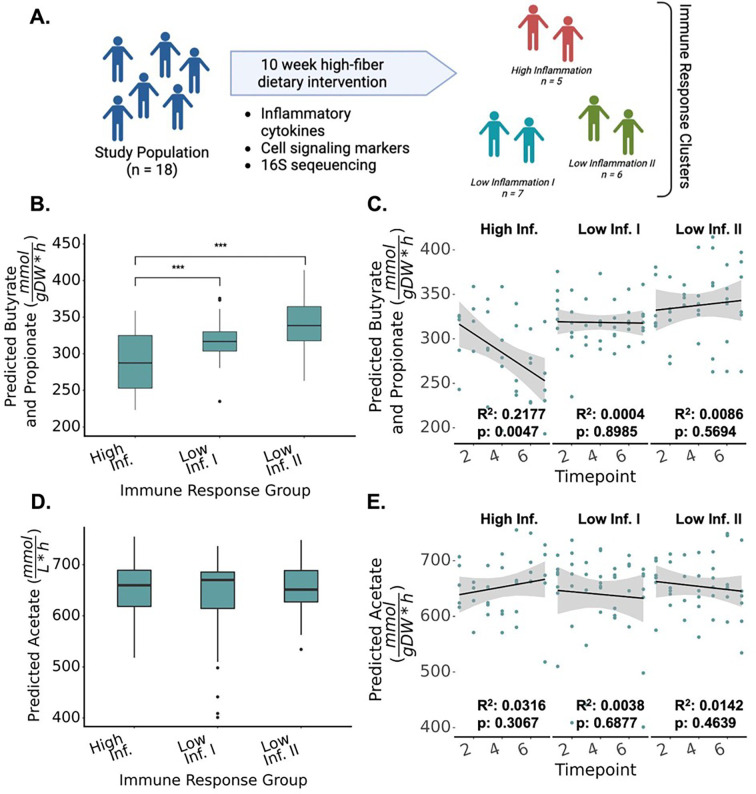
Predicted SCFA production profiles were associated with variable immune response groups following a high-fiber dietary intervention. **(A)** Summary of the study from Wastyk et al.^26^, where a cohort of 18 individuals participated in a 10-week high-fiber dietary intervention. Immune profiling based on circulating inflammatory cytokines and immune cells clustered individuals into three groups: two low-inflammation groups and one high-inflammation group. **(B)** Average predicted total butyrate and propionate production across the three immune-response groups identified in the original study. **(C)** Predicted total butyrate and propionate production rates across the duration of the intervention, stratified by immune response group **(D)** Average predicted total acetate production across the three immune response groups. **(E)** Predicted acetate production rates across the intervention, stratified by immune response group. In (A-E) stars denote significance under an Independent Student’s t-test, *** = p<0.001.

We next investigated whether MCMM-predicted SCFA production rates could be leveraged to help explain inter-individual differences in phenotypic response following a dietary intervention. Specifically, we looked at data from 18 individuals who were placed on a high-fiber diet for ten weeks ^26^. These individuals fell into three distinct immunological response groups: one in which high inflammation was observed over the course of the intervention (high-inflammation group), and two other distinct response groups that both exhibited lower levels of inflammation (low-inflammation groups I and II; Fig. 4A). We hypothesized that these immune response groups could be explained, in part, by differences in MCMM-predicted SCFA production profiles. Using 16S amplicon sequencing data from seven time points collected from each of these 18 individuals over the 10-week intervention, we built MCMMs for each study participant at each time point in the study. Growth was then simulated for each model using a standardized high-fiber diet, rich in resistant starch (see Material and Methods). Throughout the study, individuals in the high-inflammation group showed significantly lower predicted butyrate plus propionate production on average (i.e., the two SCFAs with the strongest anti-inflammatory effects), compared to the individuals in each of the low-inflammation groups (High vs. Low I: 284.6 ± 7.7 vs 327.5 ± 3.8 mmol/(gDW h) on average, Mann-Whitney p = 1.9e-5. High vs. Low II: 284.6 ± 7.7 vs 337.8 ± 6.4 mmol/(gDW h), Mann-Whitney p = 7.2e-6) (Fig. 4B). Predicted levels of butyrate plus propionate production in the high-inflammation group decreased throughout the duration of the high-fiber intervention (Pearson r = −0.47, Pearson test, p= 4.7e-3) (Fig. 4C), while predicted levels of butyrate and propionate production in the low-inflammation groups were constant over time (Low I: Pearson r = −0.020, Pearson test p= 0.90, Low II: Pearson r = 0.093, Pearson test, p = 0.57) (Fig. 4C). Acetate production did not appear to differ across groups (High vs. Low I: 652.7 ± 52.4 vs 639.8 ± 79.3 mmol/(gDW h) on average, Mann-Whitney p = .99. High vs. Low II: 652.7 ± 52.4 vs 653.8 ± 50.0 mmol/(gDW h), Mann-Whitney p = .97. Low I vs. Low II: 652.7 ± 52.4 vs 639.8 ± 79.3 mmol/(gDW h), Mann-Whitney p = .80 )(Fig. 4D), although there was a slight trend towards increasing acetate production over time in the high inflammation group (Pearson r = 0.18, p = 0.31) (Fig. 4E).

### MCMM-predicted SCFA profiles are associated with a wide range of blood-based clinical markers

**Figure 5.**
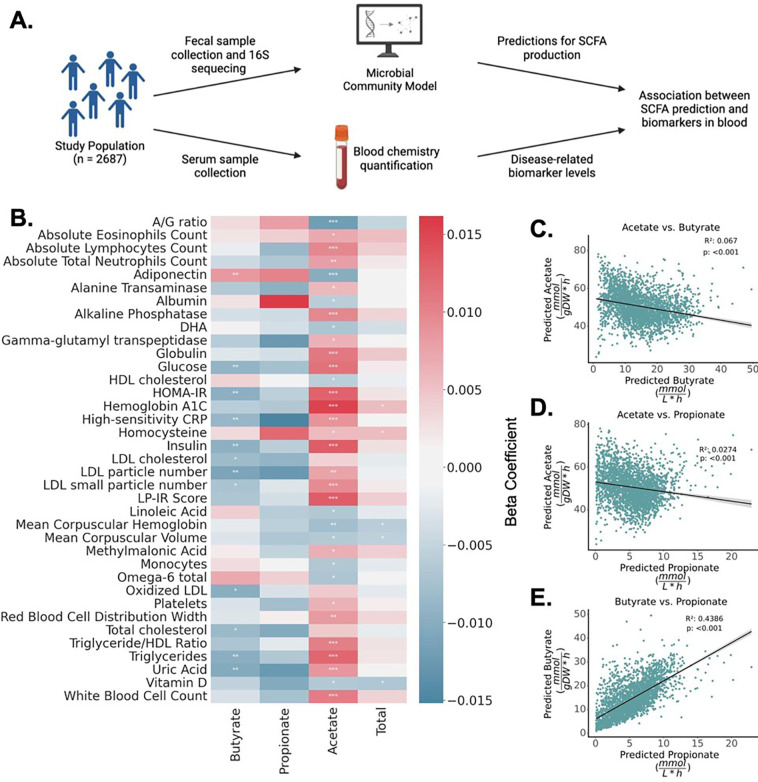
SCFA flux predictions are significantly associated with blood-derived clinical markers. **(A)** MCMMs were constructed for 2,687 Arivale participants, assuming an average European diet, to predict SCFA production profiles. SCFA predictions were regressed against a set of 128 blood-based clinical labs and health markers, with sex, age, and sequencing vendor as covariates in the regressions. **(B)** Heatmap showing the 37 significant associations (FDR-corrected Wald test p<0.05) between measured blood markers and predicted SCFA production rates. **(C-E)** Relationship between pairs of predicted SCFA production rates. Each dot denotes an individual model reconstructed for a single sample in the Arivale study (n=2,687). The black line denotes a linear regression line and the gray area denotes the 95% confidence interval of the regression. R^2^ and p-values from Pearson’s correlations.

To further evaluate the clinical relevance of personalized MCMMs, we generated SCFA production rate predictions from stool 16S amplicon sequencing data for 2,687 individuals in a deeply phenotyped, generally-healthy cohort from the West Coast of the United States (i.e., the Arivale cohort) ^27^. Baseline MCMMs were built for each individual assuming the same dietary input (i.e., an average European diet) in order to compare SCFA production rate differences, independent of background dietary variation. MCMM-predicted SCFA fluxes were then regressed against a panel of 128 clinical chemistries and health metrics collected from each individual, adjusting for a standard set of common covariates (i.e., age, sex, and microbiome sequencing vendor) (Fig. 5A). After FDR correction, 37 markers were significantly associated with the predicted production rate of at least one SCFA (Fig. 5B). Predicted butyrate production showed significant positive association with the health-associated hormone adiponectin, and significant inverse association with 11 metabolites associated with poorer health, including C-reactive protein (CRP), HOMA-IR, and low-density lipoprotein (LDL; P < 0.05, FDR-corrected Wald test). Acetate showed significant positive associations with 23 blood metabolites and inverse associations with 11 metabolites (Fig. 5B), which tended to be in the opposite direction as the butyrate associations. Propionate showed no significant associations, while overall SCFA production showed 2 positive and 3 negative associations (Fig. 5B). Butyrate and propionate production tended to be positively correlated within an individual, while higher acetate production was inversely associated with both butyrate and propionate production (Fig. 5C–D). This inverse association may be responsible, in part, for the flipped associations with clinical chemistries between butyrate and acetate.

### Leveraging MCMMs to design precision dietary, prebiotic, and probiotic interventions

**Figure 6.**
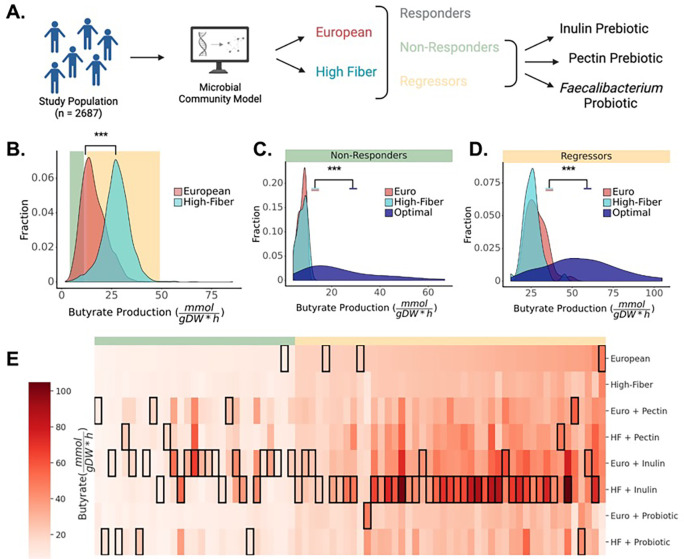
Microbial MCMMs can be used to design, build, and test personalized prebiotic, probiotic, and dietary interventions aimed at optimizing SCFA production profiles. **(A)** MCMMs built from the Arivale cohort (N = 2,687) were used to test personalized responses to dietary interventions. Personalized models were simulated on an average European (Euro) diet, as well as on a high-fiber diet, and divided into responders, non-responders, and regressors, based on the changes in predicted butyrate production in response to increasing dietary fiber. Non-responders were defined as individuals who produced less than 10 $\frac{mmol}{gDW\;*\;h}$ of butyrate on the European diet and showed an increase of less than 20% in butyrate production on the high-fiber diet. Regressors were defined as individuals who produced at least 20 $\frac{mmol}{gDW\;*\;h}$ butyrate on the European diet and showed a drop in butyrate production on the high-fiber diet. Single-fiber and probiotic interventions were applied to non-responders and regressors. **(B)** Distribution of butyrate production rates on two different diets simulated for all participants in the study. Butyrate production ranges that contain non-responders (N=29) and regressors (N=45) are highlighted in green and yellow shaded areas, respectively. **(C)** Distributions of butyrate production rates for the non-responder group (N=29). The optimal intervention resulting in the highest butyrate production is shown in blue. **(D)** Butyrate production rates for the regressor group (N=45). The optimal intervention that resulted in the highest butyrate production is shown in blue. **(E)** Heatmap of butyrate production rates across simulated interventions for the individuals in the non-responder and regressor groups. Rows denotes specific interventions (Euro - average European diet, HF - high fiber diet). Columns denote individuals in the response groups (N=74). Cell shading (white-to-red) denotes butyrate production rate. Added interventions tested on both non-responders and regressors included probiotic supplementation (inulin or pectin) as well as prebiotic supplementation (10% relative abundance *Faecalibacterium*). The most successful intervention for each individual is denoted by a black border around that cell in the corresponding column.

As a proof-of-concept for *in silico* engineering of the metabolic outputs of the human gut microbiome, we screened a set of potential interventions designed to increase SCFA production for individuals from the Arivale cohort (Fig. 6A). MCMMs were built using two different dietary contexts: an average European diet, and a vegan, high-fiber diet (see Material and Methods). Predicted butyrate production rates were then compared across the two diets. As expected, models grown on a high-fiber diet showed higher average predicted butyrate production: 27.35 ± 6.77 mmol/(gDW h) vs 16.17 ± 6.22 mmol/(gDW h), paired t-test, t = 92.74, p < .001 (Fig. 6B). However, this increase in butyrate production between the European and high-fiber diets was not uniform across individuals. On the high-fiber diet, some individual gut microbiota compositions showed very large increases in butyrate production, some showed little-to-no change, and a small subset of samples actually showed a decrease in butyrate production, relative to the European diet. We identified a set of ‘non-responders’ (n = 29) who produced less than 10 $\frac{mmol}{gDW\;*\;h}$ of butyrate on the European diet and showed an increase in butyrate production of less than 20% on the high-fiber diet (Fig. 6C). We also identified a set of ‘regressors’ (n = 45) who produced more than 20 $\frac{mmol}{gDW\;*\;h}$ of butyrate on the European diet and showed lower butyrate production on the high-fiber diet (Fig. 6D). We then simulated a handful of simple prebiotic and probiotic interventions across these individuals, to identify optimal intervention combinations for each individual (Fig. 6C–E). MCMMs for each subset of individuals were simulated with prebiotic and probiotic interventions in the context of either the European or the high-fiber diet. Specifically, diets were supplemented with the dietary fiber inulin, with the dietary fiber pectin, or with a simulated probiotic intervention that consisted of introducing 10% relative abundance of the butyrate-producing genus *Faecalibacterium* to the MCMM. In general, optimal combination interventions significantly increased the population-level butyrate production above either dietary intervention alone (Fig. 6C–D).

For 70/74 individuals, supplementation of the background diet with a specific pre- or probiotic increased the butyrate production rate (Fig. 6C–E). Neither response group had an intervention that was optimal for all individuals. In general, the most successful intervention for non-responders was the addition of inulin to the European diet (156.6% ± 183.3% increase vs standard European diet), and for regressors it was the addition of inulin to the high-fiber diet (88.2% ± 75.7% increase). However, the exact intervention that yielded the highest butyrate production for any given individual across both populations varied widely (Fig. 6E). For example, the probiotic intervention was more successful in raising predictions for butyrate production in non-responders than it was in regressors (Fig. 6E). The optimal intervention combination in the non-responder subpopulation was more heterogeneous than in the regressor subpopulation. Overall, no single intervention combination was optimal for every individual in the population.

## Discussion

Here we present an approach to the rational engineering of SCFA production rates from the human gut microbiome through prebiotic, probiotic, and dietary interventions, validated using *in vitro* and *in vivo* experimental data. We demonstrated the MCMMs can be used to formulate personalized interventions designed to optimize SCFA production profiles.

Model predictions of butyrate production in synthetically constructed *in vitro* co-cultures showed significant agreement between measured and predicted butyrate fluxes (Fig. 2). Due to the phylogenetic resolution of 16S data and the lack of strain-level GEMs that match the organisms present in some samples, we built genus-level MCMMs for all analyses. The decreasing accuracy of butyrate predictions as community richness declined may reflect a limitation of building models at the genus-level, as reconstructions contain a summarized aggregation of the metabolic capability of the genus as a whole, without species- or strain-level resolution. Consequently, pathways included in the metabolic model may be absent in a low-richness experimental system, reflecting a mismatch between the modeling framework and reality. In high richness models, predictions became more accurate, indicating this mismatch is less impactful as more taxa are included in the system. Real-world microbiomes are often more species-rich than synthetic *in vitro* communities. Fortunately, we are likely not operating in a community regime where missingness in individual models has a large influence on genus-level MCMM SCFA predictions in the human gut. However, future work should focus on increasing the availability of diverse strain-level GEMs. The recent release of AGORA2^28^, containing GEMs for 7,302 microorganisms, may help to overcome this limitation and aid in the construction of MCMMs at finer levels of taxonomic resolution.

Data from *ex vivo* anaerobic fecal incubations showed agreement between SCFA flux predictions and measurements. Fiber-treated samples showed significant increases in both predicted and measured SCFA production, compared to the controls (Fig. 3). Additionally, measured and predicted production rates of butyrate and propionate showed quantitative agreement across three independent studies. Acetate production rates were accurately predicted in all but one study. Acetate is known to act as an overflow metabolite ^29,30^, with a wide range of possible fluxes for a given biomass optimum, so it is perhaps not surprising that the predictions for this metabolite tended to be less accurate. Finally, predictions were generally the weakest in Study C (Fig. 3G–I). One possible reason for poorer predictions in this study is that the incubation time was shorter than for the other two studies (4 hours in Study C, vs 7 hours and 24 hours in Studies A and B, respectively), resulting in less divergence in accumulated SCFA concentrations between controls and treated samples. Thus, it is likely that SCFA levels did not build up to high enough levels in this study to accurately reflect *in situ* production. Overall, the observed correspondence between our SCFA production profile predictions and *in vitro* data provided us with some confidence in our MCMM modeling strategy and prompted us to explore how these predictions might be applied to an *in vivo* setting.

MCMMs built using data from a 10-week high-fiber dietary intervention allowed us to assess our predictions *in vivo* in the context of immunological responses to diet. Predictions of combined butyrate and propionate (i.e., SCFAs with the strongest anti-inflammatory effects on the host ^31^) production rates over the course of the high-fiber intervention showed distinct differences between pre-defined immune response groups (Fig. 4). The low-inflammation groups showed stable butyrate and propionate production fluxes over time, while the high inflammation group showed lower average butyrate and propionate production and a decreasing production rate over time following the initiation of the high-fiber diet (Fig. 4B–C). Given the strong anti-inflammatory effects of butyrate and propionate, we expected to see lower production of these molecules in the context of higher inflammation. Prior work has shown that higher doses of inulin can actually induce an inflammatory response, which may explain, in part, the inflammatory immune response in these individuals ^32^. Overall, our results indicate that different immunological responses to a high fiber diet may be explained, in part, by the observed heterogeneity in MCMM-predicted butyrate and propionate production rates (Fig. 4).

Several biomarkers of metabolic health, inflammation, liver function, and cardiovascular health were associated with MCMM-predicted SCFA production profiles in a large, generally-healthy cohort, assuming an average European diet (Fig. 5). CRP, a marker of systemic inflammation ^33^, showed a significant negative association with butyrate production predictions. Markers of cardiovascular health, such as LDL and triglyceride levels, were also negatively associated with butyrate production rates, supporting the role of butyrate as protective against cardiovascular disease ^34^. Many significant associations were inverted when looking across butyrate and acetate flux predictions (Fig. 5). For instance, CRP, HOMA-IR, glucose, insulin, LDL cholesterol and uric acid all showed significant negative associations with butyrate production and significant positive associations with acetate production. This result may be related to the apparent tradeoff between acetate production and the production of both butyrate and propionate (Fig. 5C–E). While the overall production of SCFAs has been implicated in lowering inflammation^35^, the potency of butyrate in driving down inflammation and improving overall metabolic health is greater than that of acetate ^36^, which, given this apparent tradeoff in the production of these different SCFAs, could help to explain these inverted associations.

Given this set of promising associations between SCFA predictions and host phenotypic variation, we next wanted to demonstrate the potential of MCMMs for designing precision prebiotic, probiotic, and dietary interventions that optimize SCFA production profiles. Using the Arivale cohort, we identified two classes of individuals that responded differently to an *in silico* high-fiber dietary intervention: non-responders and regressors (Fig. 6). We found significant heterogeneity in the optimal intervention across individuals from each of these response groups, but most notably in the non-responders (Fig. 6E). Given that the non-responders had low baseline levels of butyrate production and did not respond to a high-fiber diet, this underscores the importance of personalized predictions for those who tend not to respond well to population-scale interventions.

Personalized prediction of SCFA production profiles from human gut MCMMs represents an important technological step forward in leveraging artificial intelligence for precision nutrition. Mechanistic modeling allowed us to translate the ecological composition of the gut microbiome into concrete, individual-specific metabolic outputs, in response to specific interventions ^37^. MCMMs are transparent models that do not require training data, with clear causal and mechanistic explanations behind each prediction. Microbially-produced metabolites have an substantial impact on host physiology and health ^38,39^, and a rational framework for engineering the production or consumption rates of these metabolites has broad potential applications in precision nutrition and personalized healthcare.

## Materials and Methods

### In vitro culturing

Culturing of the synthetically assembled gut microbial communities is described in Clark et al., 2021 ^25^. Culturing of *ex vivo* samples in Study A was done using the methodology described below. Culturing of *ex vivo* samples in Study B is described in Cantu-Jungles et al., 2021^14^. Culturing of *ex vivo* samples in Study C was conducted by co-author Dr. Thomas Gurry, using the methodology described below.

### In vitro culturing of fecal-derived microbial communities (Study A)

Fecal samples were collected in 1200 mL 2-piece specimen collectors (Medline, USA) in the Public Health Science Division of the Fred Hutchinson Cancer Center (IRB Protocol number 5722) and transferred into an large vinyl anaerobic chamber (Coy, USA, 37°C, 5% hydrogen, 20% carbon dioxide, balanced with nitrogen) at the Institute for Systems Biology within 20 minutes of defecation. All further processing and sampling was then run inside the anaerobic chamber. 50 g of fecal material was transferred into sterile 50 oz Filter Whirl-Paks (Nasco, USA) with sterile PBS + 0.1% L-cysteine at a 1:2.5 w/v ratio and homogenized with a Stomacher Biomaster (Seward, USA) for 15 minutes. After homogenization, each sample was transferred into three sterile 250 mL serum bottles and another 2.5 parts of PBS + 0.1% L-cysteine was added to bring the final dilution to 1:5 in PBS. 87 ug/mL inulin or an equal volume of sterile PBS buffer were added to treatment or control bottles, respectively. Samples were immediately pipetted onto sterile round-bottom 2 mL 96-well plates in triplicates. Baseline samples were aliquoted into sterile 1.5 mL Eppendorf tubes and the plates were covered with Breathe-Easy films (USA Scientific Inc., USA). Plates were incubated for 7 h at 37°C and gently vortexed every hour within the chamber. Final samples were aliquoted into 1.5 mL Eppendorf tubes at the end of incubation. Baseline and 7 h samples were kept on ice and immediately processed after sampling. 500 uL of each sample were aliquoted for metagenomics and kept frozen at −80°C before and during transfer to the commercial sequencing service (Diversigen, Inc). The remaining sample was transferred to a table-top centrifuge (Fisher Scientific accuSpin, USA) and spun at 1,500 rpm for 10 minutes. The supernatant was then transferred to collection tubes kept on dry ice and transferred to the commercial metabolomics provider Metabolon, USA, for targeted SCFA quantification.

### In vitro culturing of fecal-derived microbial communities (Study C)

Homogenized fecal samples in this study again underwent anaerobic culturing at 37°C, as described above, but with a shorter culturing time of 4 hours. The slurry was diluted 2.5x in 0.1% L-cysteine PBS buffer solution. Cultures were supplemented with the dietary fibers pectin or inulin to a final concentration of 10g/L, or a sterile PBS buffer control treatment. Aliquots were taken at 0h and 4h and further processed for measurement of SCFA concentrations, which were used to estimate experimental production flux (concentration[4h] - concentration[0h]/4h). SCFA concentrations were measured using GC-FID. Briefly, the pH of the aliquots was adjusted to 2–3 with 1% aqueous sulfuric acid solution, after which they were vortexed for 10 minutes and centrifuged for 10 minutes at 10,000 rpm. 200 uL aliquots of clear supernatant were transferred to vials containing 200 uL of MeCN and 100 uL of a 0.1% v/v 2-methyl pentanoic acid solution. The resulting solutions were analyzed by GC-FID on a Perkin Elmer Clarus 500 equipped with a DB-FFAP column (30m, 0.250mm diameter, 0.25um film) and a flame ionization detector.

### Metagenomic sequencing and analysis

For Study A, shallow metagenomic sequencing was performed by the sequencing vendor Diversigen, USA (i.e., their BoosterShot service). In brief, DNA was extracted from the fecal slurries with the DNeasy PowerSoil Pro Kit on a QiaCube HT (Qiagen, Germany) and quantified using the Qiant-iT Picogreen dsDNA Assay (Invitrogen, USA). Library preparation was performed with a proprietary protocol based on the Nextera Library Prep kit (Illumina, USA) and the generated libraries were sequenced on a NovaSeq (Illumina, USA) with a single-end 100bp protocol. Demultiplexing was performed using Illumina BaseSpace to generate the final FASTQ files used during analysis.

Preprocessing of raw sequencing reads was performed using FASTP ^40^. The first 5bp on the 5’ end of each read were trimmed, and the 3’ end was trimmed using a sliding window quality filter that would trim the read as soon as the average window quality fell below 20. Reads containing ambiguous base calls or with a length of less than 15bp after trimming were removed from the analysis.

Bacterial species abundances were quantified using Kraken2 v2.0.8 and Bracken v2.2 using the Kraken2 default database which was based on Refseq release 94, retaining only those species with at least 10 assigned reads ^41,42^. The analysis pipeline can be found at https://github.com/Gibbons-Lab/pipelines/tree/master/shallow_shotgun.

### Metabolomics

Targeted metabolomics were performed using Metabolon’s high-performance liquid chromatography (HPLC)–mass spectrometry (MS) platform, as described before ^43^. In brief, fecal supernatants were thawed on ice, proteins were removed using aqueous methanol extraction, and organic solvents were removed with a TurboVap (Zymark, USA). Mass spectroscopy was performed using a Waters ACQUITY ultra-performance liquid chromatography (UPLC) and Thermo Scientific Q-Exactive high resolution/accuracy mass spectrometer interfaced with a heated electrospray ionization (HESI-II) source and an Orbitrap mass analyzer operated at 35,000 mass resolution. For targeted metabolomics ultra-pure standards of the desired short-chain fatty acids were used for absolute quantification. Fluxes for individual metabolites were estimated as the rate of change of individual metabolites during the incubation period (concentration[7h] - concentration[0h]/7h).

### Model Construction

Taxonomic abundance data summarized to the genus level, inferred from 16S amplicon sequencing or shotgun metagenomic sequencing, were used to construct all MCMMs in this analysis using the community-scale metabolic modeling platform MICOM v0.32.3 ^17^. Models were built using the MICOM build() function with a relative abundance threshold of 0.001, omitting taxa that made up less than 0.1% relative abundance. The AGORA database (v1.03) of taxonomic reconstructions summarized to the genus level was used to collect genome-scale metabolic models for taxa present in each model. *In silico* media were applied to the grow() function, defining the bounds for metabolic imports by the MCMM. Medium composition varied between analyses (see Media Construction). Steady state growth rates and fluxes for all samples were then inferred using cooperative tradeoff flux balance analysis (ctFBA). In brief, this is a two-step optimization scheme, where the first step finds the largest possible biomass production rate for the full microbial community and the second step infers taxon-specific growth rates and fluxes, while maintaining community growth within a fraction of the theoretical maximum (i.e., the tradeoff parameter), thus balancing individual growth rates and the community-wide growth rate ^17^. For all models in the manuscript we used a tradeoff parameter of 0.7. This parameter value was chosen through cooperative tradeoff analysis in MICOM. Multiple parameters were tested, and the highest parameter value (i.e., the value closest to the maximal community growth rate at 1.0) that allowed most (>90%) of taxa to grow was chosen (i.e., 0.7)*.* Predicted growth rates from the simulation were analyzed to validate correct behavior of the models. All models were found to grow with minimum community growth rate of 0.3 h^−1^. Predicted values for export fluxes of SCFAs were collected from each MCMM using the production_rates() function, which calculates the overall production from the community that would be accessible to the colonic epithelium.

### Media Construction

Individual media were constructed based on the context of each individual analysis. For the synthetic *in vitro* cultures conducted by Clark et al. (2021), a defined medium (DM38) was used that supported growth of all taxa used in the experiments, excluding *Faecalibacterium prausnitzii.* Manually mapping each component to the Virtual Metabolic Human database, we constructed an *in silico* medium with flux bounds scaled to component concentration. All metabolites were found in the database. Using the MICOM fix_medium() function, a minimal set of metabolites necessary for all models to grow to a minimum community growth rate of 0.3 h^−1^ was added to the medium - here, only iron(III) was added (*in silico* medium available here: https://github.com/Gibbons-Lab/scfa_predictions/tree/main/media).

To mimic the medium used in *ex vivo* cultures of fecally-derived microbial communities, a diluted, carbon-stripped version of a standard European diet was used. First, a standard European diet was collected from the Virtual Metabolic Human database (www.vmh.life/#nutrition) ^44^. Components in the medium which could be imported by the host, as defined by an existing uptake reaction in the Recon3D model ^45^, were diluted to 20% of their original flux, to adjust for absorption in the small intestine^45^. Additionally, host-supplied metabolites such as mucins and bile acids were added to the medium. As most carbon sources are consumed in the body and are likely not present in high concentrations in stool, this diet was then algorithmically stripped of carbon sources by removing metabolites with greater than six carbons and no nitrogen, to avoid removing nitrogen sources. Additionally, the remaining metabolites in the medium were diluted to 10% of their original flux, mimicking the nutrient-depleted fecal homogenate. This medium was also augmented using the fix_medium() function in MICOM. To simulate fiber supplementation, single fiber additions were made to the medium, either pectin (0.75 mmol/gDW*h) or inulin (10.5 mmol/gDW*h). Bounds for fiber supplementation were chosen to balance the carbon content of each, as represented in the model (pectin: 2535 carbons, inulin: 180 carbons).

For *in vivo* modeling, two diets were used: a high-fiber diet containing high levels of resistant starch, and a standard European diet ^44,46^. Again, both diets were collected from the Virtual Metabolic Human database (www.vmh.life/#nutrition). Each medium was subsequently adjusted to account for absorption in the small intestine by diluting metabolite flux as described previously. Additionally, host-supplied metabolites such as mucins and bile acids were added to the medium, to match the composition of the medium *in vivo*. Finally, the complete_medium() function was again used to augment the medium, as described above.

Prebiotic interventions were designed by supplementing the high-fiber or average European diet with single fiber additions, either pectin or inulin. As before, bounds for fiber addition were set as 0.75 mmol/gDW*h for pectin and 10.5 mmol/gDW*h for inulin.

### Probiotic Intervention

To model a probiotic intervention, 10% relative abundance of the genus *Faecalibacterium*, a known butyrate-producing taxon ^47^, was added to the MCMMs by adding a pan-genus model of the taxon derived from the AGORA database version 1.03. Measured taxonomic abundances were scaled to 90% of their initial values, after which *Faecalibacterium* was artificially added to the model.

### External Data Collection

Data containing taxonomic abundance, optical density, and endpoint butyrate concentration for synthetically-constructed *in vitro* microbial cultures were collected from Clark et al. (2021) ^25^. Endpoint taxonomic abundance data, calculated from fractional read counts collected via 16S amplicon sequencing, was used to construct individual MCMMs for each co-culture (see Model Construction). Resulting models ranged in taxonomic richness from 1 to 25 taxa.

From a second study by Cantu-Jungles et al. (2021) ^14^ (*ex vivo* Study B), preprocessed taxonomic abundance and SCFA metabolomics data was collected. Homogenized fecal samples in this study underwent a similar culturing process, with a culturing time of 24 hours. Cultures were supplemented with the dietary fiber pectin, or a PBS control. Initial and endpoint metabolomic SCFA measurements were used to estimate experimental production flux (concentration[24h] - concentration[0h]/24h). Taxonomic abundance data was used to construct MCMMs for each individual (see Model Construction).

Data from a third (Study C) was collected from the Pharmaceutical Biochemistry Group at the University of Geneva, Switzerland, under study protocol 2019–00632, containing sequencing data in FASTQ format and targeted metabolomics SCFA measurements.

Data was collected from Wastyk, et al 2021 ^26^, which provided 16S amplicon sequencing data at 9 timepoints spanning 14 weeks, along with immunological phenotyping, for 18 participants undergoing a high-fiber dietary intervention. Only 7 timepoints spanning 10 weeks were included in subsequent analysis, as the last 2 timepoints were taken after the conclusion of the dietary intervention. MCMMs were constructed for each participant at each timepoint at the genus level (see Model Construction). Mean total butyrate and propionate production, as well as acetate production, were compared between immune response groups.

De-identified data was obtained from a former scientific wellness program run by Arivale, Inc. (Seattle, WA) ^27^. Arivale closed its operations in 2019. Taxonomic abundances, inferred from 16S amplicon sequencing data, for 2,687 research-consenting individuals were collected and used to construct MCMMs. 128 paired blood-based clinical chemistries taken within 30 days of fecal sampling were also collected and used to find associations between MCMM SCFA predictions on a standard European diet and clinical markers.

### Statistical analysis

Statistical analysis was performed using SciPy (v1.9.1) and statsmodels (v0.14.0) in Python (v3.8.13). Pearson correlation coefficients and p-values were calculated between measured and predicted SCFA production fluxes in *in vitro* cultures*,* as well as for predicted SCFA production fluxes across timepoints for an *in vivo* high-fiber intervention. Significance in overall SCFA production between immune response groups in the high-fiber intervention was determined by pairwise Mann-Whitney U test for butyrate+propionate production and for acetate production. Association of MCMM-predicted SCFA production flux with paired blood-based clinical labs was tested using OLS regression, adjusting for age, sex, microbiome sequencing vendor, and clinical lab vendor, and tested for significance by two-sided Wald test. BMI was not included as a confounder in the analysis because it was itself negatively correlated with butyrate production ^48^. Multiple comparison correction for p-values was done using the Benjamini–Hochberg method for adjusting the False Discovery Rate (FDR) ^49^. Comparison of butyrate production between dietary interventions was tested using paired Student’s t-tests. In all analyses, significance was considered at the p<0.05 threshold.

## References

[1] Oliphant K. & Allen-Vercoe E. Macronutrient metabolism by the human gut microbiome: major fermentation by-products and their impact on host health. Microbiome 7, 91 (2019). 31196177 10.1186/s40168-019-0704-8 PMC6567490

[2] Rackerby B., Van De Grift D., Kim J. H. & Park S. H. Effects of Diet on Human Gut Microbiome and Subsequent Influence on Host Physiology and Metabolism. Gut Microbiome and Its Impact on Health and Diseases 63–84 Preprint at 10.1007/978-3-030-47384-6_3 (2020).

[3] Tomasova L., Grman M., Ondrias K. & Ufnal M. The impact of gut microbiota metabolites on cellular bioenergetics and cardiometabolic health. Nutr. Metab. 18, 72 (2021). 10.1186/s12986-021-00598-5 PMC8281717 34266472

[4] Glotfelty L. G., Wong A. C. & Levy M. Small molecules, big effects: microbial metabolites in intestinal immunity. Am. J. Physiol. Gastrointest. Liver Physiol. 318, G907–G911 (2020). 32249590 10.1152/ajpgi.00263.2019 PMC7395478

[5] Donia M. S. & Fischbach M. A. HUMAN MICROBIOTA. Small molecules from the human microbiota. Science 349, 1254766 (2015). 26206939 10.1126/science.1254766 PMC4641445

[6] Diener C. Genome-microbiome interplay provides insight into the determinants of the human blood metabolome. Nat Metab 4, 1560–1572 (2022). 36357685 10.1038/s42255-022-00670-1 PMC9691620

[7] Ríos-Covián D. Intestinal Short Chain Fatty Acids and their Link with Diet and Human Health. Front. Microbiol. 7, 185 (2016). 26925050 10.3389/fmicb.2016.00185 PMC4756104

[8] Nogal A., Valdes A. M. & Menni C. The role of short-chain fatty acids in the interplay between gut microbiota and diet in cardio-metabolic health. Gut Microbes 13, 1–24 (2021). 10.1080/19490976.2021.1897212 PMC8007165 33764858

[9] Silva Y. P., Bernardi A. & Frozza R. L. The Role of Short-Chain Fatty Acids From Gut Microbiota in Gut-Brain Communication. Frontiers in Endocrinology vol. 11 Preprint at 10.3389/fendo.2020.00025 (2020). PMC7005631 32082260

[10] Morrison D. J. & Preston T. Formation of short chain fatty acids by the gut microbiota and their impact on human metabolism. Gut Microbes 7, 189–200 (2016). 26963409 10.1080/19490976.2015.1134082 PMC4939913

[11] Cong J., Zhou P. & Zhang R. Intestinal Microbiota-Derived Short Chain Fatty Acids in Host Health and Disease. Nutrients 14, (2022). 10.3390/nu14091977 PMC9105144 35565943

[12] Tan J. The role of short-chain fatty acids in health and disease. Adv. Immunol. 121, 91–119 (2014). 24388214 10.1016/B978-0-12-800100-4.00003-9

[13] Mortensen P. B. & Clausen M. R. Short-chain fatty acids in the human colon: relation to gastrointestinal health and disease. Scand. J. Gastroenterol. Suppl. 216, 132–148 (1996). 10.3109/00365529609094568 8726286

[14] Cantu-Jungles T. M. Dietary Fiber Hierarchical Specificity: the Missing Link for Predictable and Strong Shifts in Gut Bacterial Communities. MBio 12, e0102821 (2021). 34182773 10.1128/mBio.01028-21 PMC8262931

[15] Healey G. R., Murphy R., Brough L., Butts C. A. & Coad J. Interindividual variability in gut microbiota and host response to dietary interventions. Nutr. Rev. 75, 1059–1080 (2017). 29190368 10.1093/nutrit/nux062

[16] Boets E. Quantification of in Vivo Colonic Short Chain Fatty Acid Production from Inulin. Nutrients 7, 8916–8929 (2015). 26516911 10.3390/nu7115440 PMC4663568

[17] Diener C., Gibbons S. M. & Resendis-Antonio O. MICOM: Metagenome-Scale Modeling To Infer Metabolic Interactions in the Gut Microbiota. mSystems 5, (2020). 10.1128/mSystems.00606-19 PMC6977071 31964767

[18] van Deuren T., Blaak E. E. & Canfora E. E. Butyrate to combat obesity and obesity-associated metabolic disorders: Current status and future implications for therapeutic use. Obes. Rev. 23, e13498 (2022). 35856338 10.1111/obr.13498 PMC9541926

[19] Zeevi D. Personalized Nutrition by Prediction of Glycemic Responses. Cell 163, 1079–1094 (2015). 26590418 10.1016/j.cell.2015.11.001

[20] Rein M. Effects of personalized diets by prediction of glycemic responses on glycemic control and metabolic health in newly diagnosed T2DM: a randomized dietary intervention pilot trial. BMC Med. 20, 56 (2022). 35135549 10.1186/s12916-022-02254-y PMC8826661

[21] Gibbons S. M. Perspective: Leveraging the Gut Microbiota to Predict Personalized Responses to Dietary, Prebiotic, and Probiotic Interventions. Adv. Nutr. 13, 1450–1461 (2022). 35776947 10.1093/advances/nmac075 PMC9526856

[22] Heinken A. Genome-scale metabolic reconstruction of 7,302 human microorganisms for personalized medicine. Nat. Biotechnol. (2023) doi:10.1038/s41587-022-01628-0. PMC10497413 36658342

[23] Abdill R. J., Adamowicz E. M. & Blekhman R. Public human microbiome data are dominated by highly developed countries. PLoS Biol. 20, e3001536 (2022). 35167588 10.1371/journal.pbio.3001536 PMC8846514

[24] Magnúsdóttir S. Generation of genome-scale metabolic reconstructions for 773 members of the human gut microbiota. Nat. Biotechnol. 35, 81–89 (2017). 27893703 10.1038/nbt.3703

[25] Clark R. L. Design of synthetic human gut microbiome assembly and butyrate production. Nat. Commun. 12, 3254 (2021). 34059668 10.1038/s41467-021-22938-y PMC8166853

[26] Wastyk H. C. Gut-microbiota-targeted diets modulate human immune status. Cell 184, 4137–4153.e14 (2021). 34256014 10.1016/j.cell.2021.06.019 PMC9020749

[27] Manor O. Health and disease markers correlate with gut microbiome composition across thousands of people. Nat. Commun. 11, 5206 (2020). 33060586 10.1038/s41467-020-18871-1 PMC7562722

[28] Heinken A. AGORA2: Large scale reconstruction of the microbiome highlights wide-spread drug-metabolising capacities. bioRxiv 2020.11.09.375451 (2020) doi:10.1101/2020.11.09.375451.

[29] Valgepea K. Systems biology approach reveals that overflow metabolism of acetate in Escherichia coli is triggered by carbon catabolite repression of acetyl-CoA synthetase. BMC Syst. Biol. 4, 166 (2010). 21122111 10.1186/1752-0509-4-166 PMC3014970

[30] Wolfe A. J. The acetate switch. Microbiol. Mol. Biol. Rev. 69, 12–50 (2005). 15755952 10.1128/MMBR.69.1.12-50.2005 PMC1082793

[31] Li M. Pro- and anti-inflammatory effects of short chain fatty acids on immune and endothelial cells. Eur. J. Pharmacol. 831, 52–59 (2018). 29750914 10.1016/j.ejphar.2018.05.003

[32] Arifuzzaman M. Inulin fibre promotes microbiota-derived bile acids and type 2 inflammation. Nature 611, 578–584 (2022). 36323778 10.1038/s41586-022-05380-y PMC10576985

[33] Sproston N. R. & Ashworth J. J. Role of C-Reactive Protein at Sites of Inflammation and Infection. Front. Immunol. 9, 754 (2018). 29706967 10.3389/fimmu.2018.00754 PMC5908901

[34] Amiri P. Role of Butyrate, a Gut Microbiota Derived Metabolite, in Cardiovascular Diseases: A comprehensive narrative review. Front. Pharmacol. 12, 837509 (2021). 35185553 10.3389/fphar.2021.837509 PMC8847574

[35] Vinolo M. A. R., Rodrigues H. G., Nachbar R. T. & Curi R. Regulation of inflammation by short chain fatty acids. Nutrients 3, 858–876 (2011). 22254083 10.3390/nu3100858 PMC3257741

[36] Tedelind S., Westberg F., Kjerrulf M. & Vidal A. Anti-inflammatory properties of the short-chain fatty acids acetate and propionate: a study with relevance to inflammatory bowel disease. World J. Gastroenterol. 13, 2826–2832 (2007). 17569118 10.3748/wjg.v13.i20.2826 PMC4395634

[37] Gurry T., Nguyen L. T. T., Yu X. & Alm E. J. Functional heterogeneity in the fermentation capabilities of the healthy human gut microbiota. PLoS One 16, e0254004 (2021). 34288919 10.1371/journal.pone.0254004 PMC8294568

[38] Gasaly N., de Vos P. & Hermoso M. A. Impact of Bacterial Metabolites on Gut Barrier Function and Host Immunity: A Focus on Bacterial Metabolism and Its Relevance for Intestinal Inflammation. Front. Immunol. 12, 658354 (2021). 34122415 10.3389/fimmu.2021.658354 PMC8187770

[39] Agus A., Clément K. & Sokol H. Gut microbiota-derived metabolites as central regulators in metabolic disorders. Gut 70, 1174–1182 (2021). 33272977 10.1136/gutjnl-2020-323071 PMC8108286

[40] Chen S., Zhou Y., Chen Y. & Gu J. fastp: an ultra-fast all-in-one FASTQ preprocessor. Bioinformatics 34, i884–i890 (2018). 30423086 10.1093/bioinformatics/bty560 PMC6129281

[41] Wood D. E., Lu J. & Langmead B. Improved metagenomic analysis with Kraken 2. Genome Biol. 20, 257 (2019). 31779668 10.1186/s13059-019-1891-0 PMC6883579

[42] Lu J., Breitwieser F. P., Thielen P. & Salzberg S. L. Bracken: estimating species abundance in metagenomics data. PeerJ Comput. Sci. 3, e104 (2017). 10.7717/peerj-cs.104 PMC12016282 40271438

[43] Gauglitz J. M. Enhancing untargeted metabolomics using metadata-based source annotation. Nat. Biotechnol. 40, 1774–1779 (2022). 35798960 10.1038/s41587-022-01368-1 PMC10277029

[44] Elmadfa I. Österreichischer Ernährungsbericht 2012. 1, (2012).

[45] Brunk E. Recon3D enables a three-dimensional view of gene variation in human metabolism. Nat. Biotechnol. 36, 272–281 (2018). 29457794 10.1038/nbt.4072 PMC5840010

[46] Waldmann A., Koschizke J. W., Leitzmann C. & Hahn A. Dietary intakes and lifestyle factors of a vegan population in Germany: results from the German Vegan Study. Eur. J. Clin. Nutr. 57, 947–955 (2003). 12879089 10.1038/sj.ejcn.1601629

[47] Zhou L. Faecalibacterium prausnitzii Produces Butyrate to Maintain Th17/Treg Balance and to Ameliorate Colorectal Colitis by Inhibiting Histone Deacetylase 1. Inflamm. Bowel Dis. 24, 1926–1940 (2018). 29796620 10.1093/ibd/izy182

[48] Coppola S., Avagliano C., Calignano A. & Berni Canani R. The Protective Role of Butyrate against Obesity and Obesity-Related Diseases. Molecules 26, (2021). 10.3390/molecules26030682 PMC7865491 33525625

[49] Benjamini Y. & Hochberg Y. Controlling the false discovery rate: A practical and powerful approach to multiple testing. J. R. Stat. Soc. 57, 289–300 (1995).

