# Supplementary material for: Microbial community-scale metabolic modeling predicts personalized short chain fatty acid production profiles in the human gut
Source: bioRxiv. 2023 Nov 28:2023.02.28.530516. Originally published 2023 Mar 1. Preprint. [Version 2] doi: 10.1101/2023.02.28.530516 (PMC10002715; doi:10.1101/2023.02.28.530516)
Supplement: 1 [file NIHPP2023.02.28.530516V2-supplement-1.pdf]

bioRxiv preprint doi: https://doi.org/10.1101/2023.02.28.530516; this version posted November 28, 2023. The copyright holder for this preprint (which was not certified by peer review) is the author/funder, who has granted bioRxiv a license to display the preprint in perpetuity. It is made available under aCC-BY 4.0 International license.

Supplemental Figures and Captions

bioRxiv preprint doi: https://doi.org/10.1101/2023.02.28.530516; this version posted November 28, 2023. The copyright holder for this preprint (which was not certified by peer review) is the author/funder, who has granted bioRxiv a license to display the preprint in perpetuity. It is made available under aCC-BY 4.0 International license.

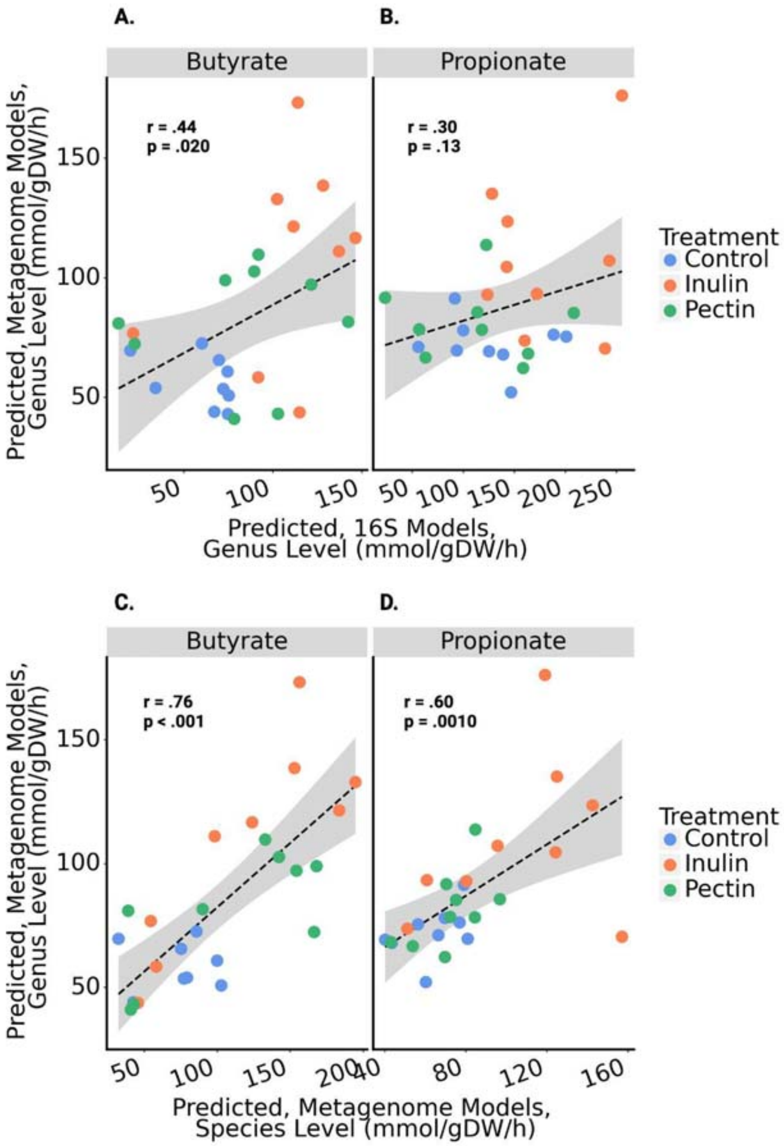

Figure S1. Predictions of SCFA production using 16S amplicon sequencing or shotgun metagenomic sequencing data show concordance. Data from Study C included 16S amplicon sequencing as well as shotgun metagenomic sequencing. The black line denotes a linear regression line and the gray area denotes the 95% confidence interval of the regression. Color encoding indicates the specific fiber treatment given to each sample. **(A-B)** Predictions for butyrate and propionate between models summarized to the genus level from 16S amplicon sequencing data and shotgun metagenome data. **(C-D)** Predictions for butyrate and propionate from models built using shotgun metagenome data at the genus level and species level.

bioRxiv preprint doi: https://doi.org/10.1101/2023.02.28.530516; this version posted November 28, 2023. The copyright holder for this preprint (which was not certified by peer review) is the author/funder, who has granted bioRxiv a license to display the preprint in perpetuity. It is made available under aCC-BY 4.0 International license.

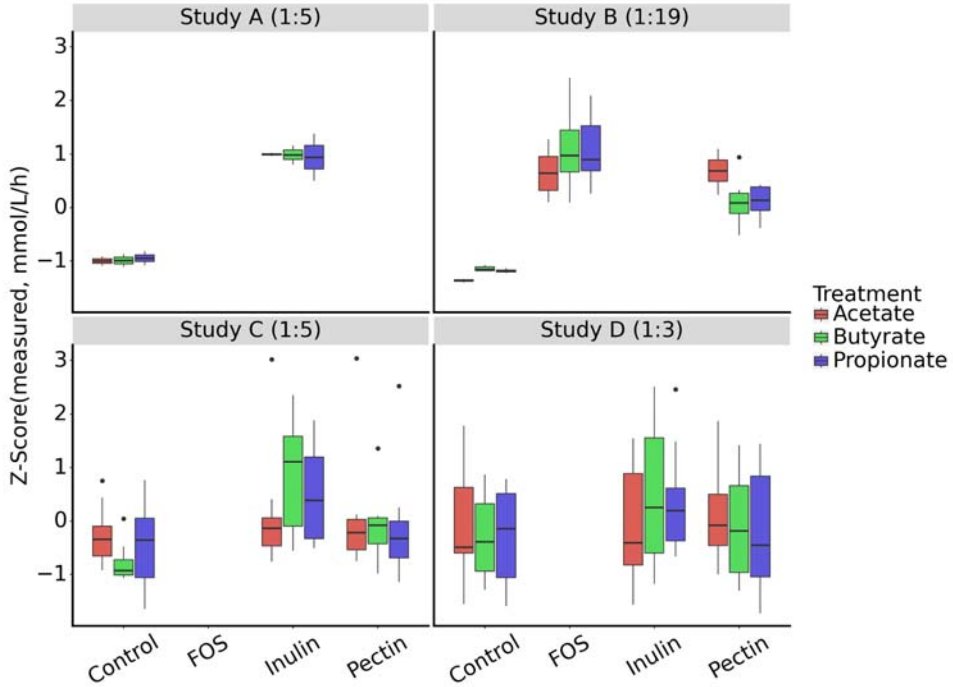

Figure S2. Divergence in SCFA production between controls and fiber-treated samples is related to culture dilution. Four independent *ex vivo* studies were used to validate predictions of MCMMs. Each study used a different dilution for the final culture, changing the scale of substrates available to the microbial communities. Illustrated here, the dilution factor, shown next to the study name, seems to show agreement with the divergence in SCFA production between control samples and fiber-treated samples. This was accounted for by diluting the residual fiber available to the microbial communities in the *in silico* medium.

bioRxiv preprint doi: https://doi.org/10.1101/2023.02.28.530516; this version posted November 28, 2023. The copyright holder for this preprint (which was not certified by peer review) is the author/funder, who has granted bioRxiv a license to display the preprint in perpetuity. It is made available under aCC-BY 4.0 International license.

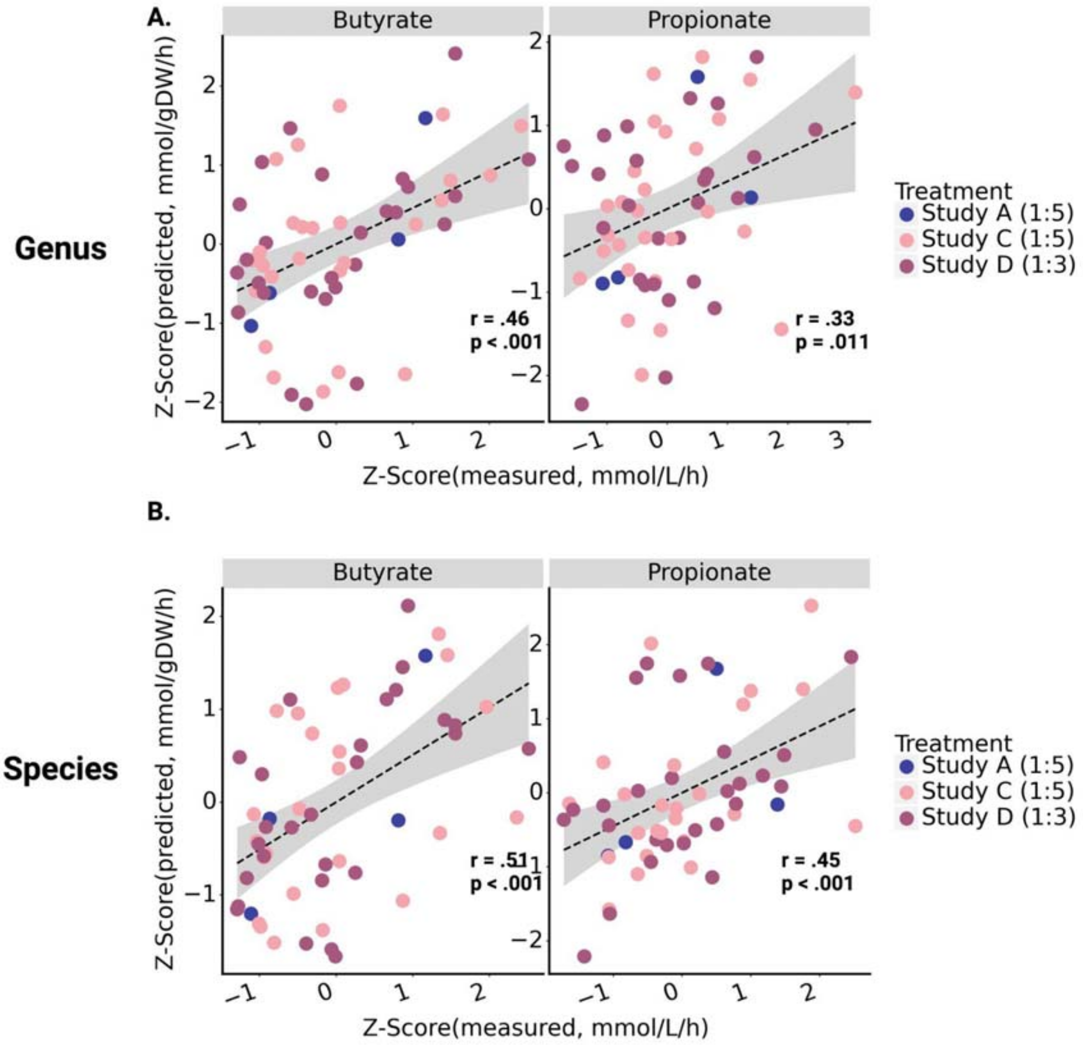

Figure S3. MCMMs built from shotgun metagenomic sequencing data perform better when constructed at the species level, as compared to the genus level. MCMMs from *ex vivo* studies A, C and D were constructed at the **(A)** genus and **(B)** species level. Prediction production rate of butyrate and propionate more closely matched measured production rate in the species level model as compared to the genus level model. The black line denotes a linear regression line and the gray area denotes the 95% confidence interval of the regression. Color encoding indicates the specific treatment from which Pearson r and associated p-value were calculated for each panel.

bioRxiv preprint doi: https://doi.org/10.1101/2023.02.28.530516; this version posted November 28, 2023. The copyright holder for this preprint (which was not certified by peer review) is the author/funder, who has granted bioRxiv a license to display the preprint in perpetuity. It is made available under aCC-BY 4.0 International license.

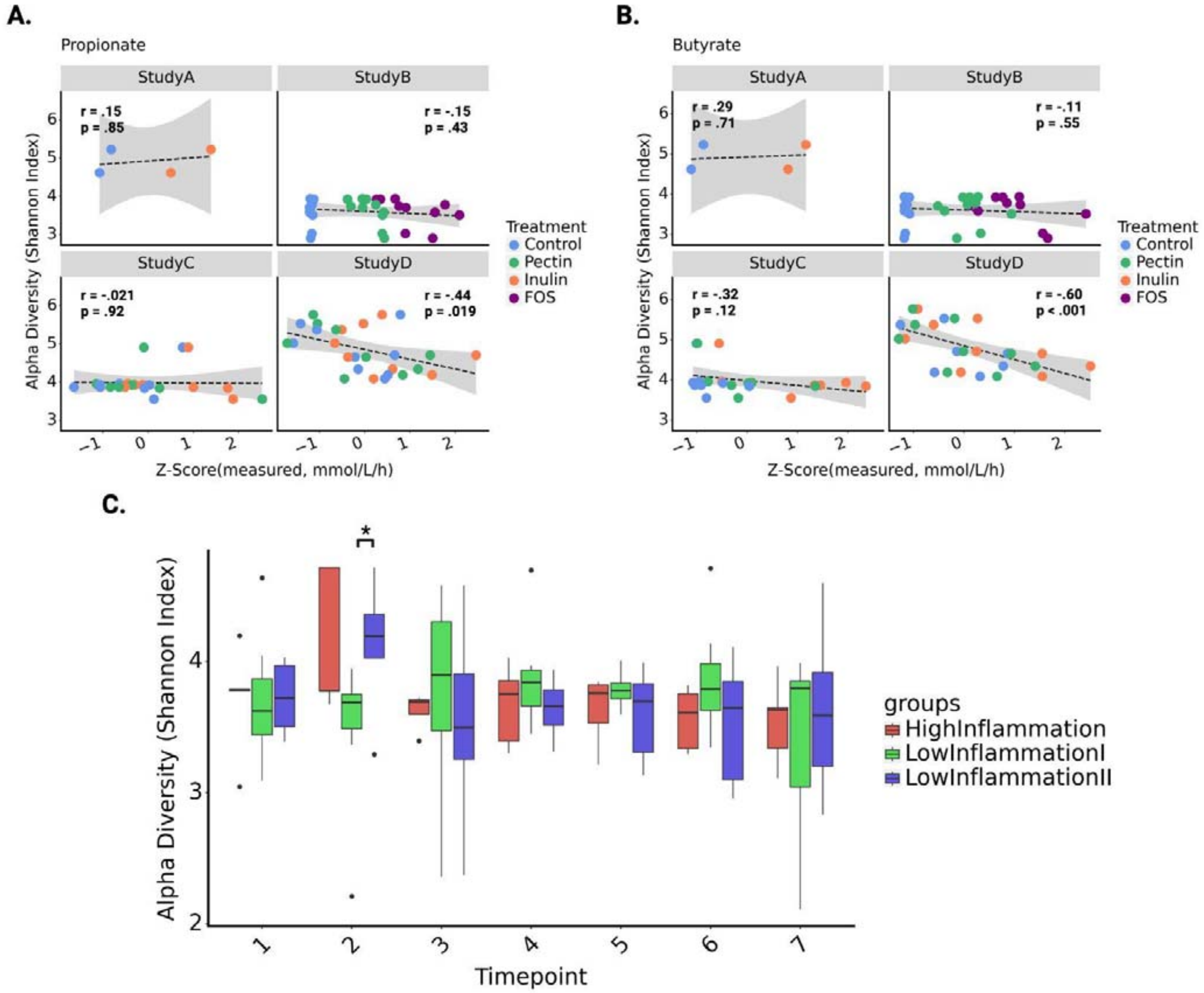

Figure S4. Alpha diversity of communities does not account for differences in SCFA production. We compared Shannon index, a measure of alpha diversity, against SCFA production in *ex vivo* communities, as well as between immune response groups in a longitudinal high fiber study. **(A)** Propionate production in four *ex vivo* datasets was not consistently explained by alpha diversity. In study D, a significant relationship was observed as determined by t-test ($p < 0.05$), but this was not consistent between datasets. The black line denotes a linear regression line and the gray area denotes the 95% confidence interval of the regression. Color encoding denotes the specific fiber treatment that was given to each sample **(B)** Butyrate production also showed no consistent correlation with alpha diversity, although a significant difference was again observed within Study D as determined by t-test ($p < 0.05$). **(C)** No consistent pattern emerged with regard to alpha diversity between immune response groups throughout the course of the high fiber dietary intervention, as determined by Mann Whitney U test for significance. In C, * = $p < 0.05$.
